# Lactate promotes the growth of patient-derived organoids from hepatopancreatobiliary cancers via ENO1/HIF1α pathway and does not affect their drug sensitivities

**DOI:** 10.1038/s41420-022-01014-4

**Published:** 2022-04-20

**Authors:** Zhiwei Wang, Yuanquan Yu, Peiyao Wu, Qinghuang Ye, Yinghao Guo, Xiaoxiao Zhang, Longfu Xi, Qi Li, Yun Jin, Donger Zhou, Yan Luo, Shuyou Peng, Jiangtao Li

**Affiliations:** 1 Department of Surgery, the Second Affiliated Hospital, Zhejiang University School of Medicine, 310009 Hangzhou, Zhejiang Province China; 2 Gastroenterology Endoscopy Center, Jiangsu Province Hospital of Chinese Medicine, Affiliated Hospital of Nanjing University of Chinese Medicine, 210029 Nanjing, Jiangsu Province China; 3 Department of Biochemistry and Cancer Institute of the Second Affiliated Hospital (Key Laboratory of Cancer Prevention and Intervention of China National MOE), Zhejiang University School of Medicine, Hangzhou, Zhejiang China

**Keywords:** Hepatocellular carcinoma, Bile duct cancer, Pancreatic cancer, Cell growth

## Abstract

The long culture duration of patient-derived organoids (PDOs) have severely limited their clinical applications. The aim of this study was to determine the effect of lactate supplementation on the growth, genetic profiles and drug sensitivities of PDOs from hepatopancreatobiliary tumors. LM3, Huh7, Panc02, and RBE cell lines were cultured as organoids in the presence or absence of lactate, and total protein was extracted to measure the expression of α-enolase (ENO1), hypoxia-inducible factor-1α (HIF1α), AKT, and PI3 kinase (PI3K). Thirteen hepatopancreatobiliary tumor specimens were collected during surgical resection and cultured as PDOs with or without l-lactate. Hematoxylin and eosin (H&E) staining and immunohistochemical staining were performed on the original tissues and PDOs to compare their pathological structures, and their genetic profiles were analyzed by whole-exome sequencing (WES). The sensitivity of the PDOs to gemcitabine, 5-fluorouracil, cisplatin, paclitaxel, ivosidenib, infigratinib, and lenvatinib were evaluated in terms of cell viability. Peripheral blood mononuclear cells (PBMCs) were isolated and co-cultured with PDOs to test the sensitivity of PDOs to tislelizumab. The addition of 20 mM lactate significantly promoted the growth of LM3 and Huh 7 organoids by 217% and 36%, respectively, compared to the control group, and the inhibition of lactate transporter decreased their growth. The HIF1α/ENO1/AKT/PI3K pathway was also activated by lactate. The inhibition of enolase also partly decreased the growth of organoids treated with lactate. Furthermore, 20 mM lactate increased the viability of 9 PDOs from 135% to 317% without affecting their pathological features. The genetic similarity, in terms of single nucleotide variations, insertions, and deletions, between original tissues and lactate-treated PDOs ranged from 83.2% to 94.1%, and that between the untreated and lactate-treated PDOs was at least 93.2%. Furthermore, the addition of lactate did not significantly change the dose–response curves of the PDOs to chemotherapeutic drugs, targeted drugs, and immune checkpoint inhibitor, especially for the drugs to which the cells were sensitive. Thus, lactate can be added to the culture medium of PDOs to promote their growth without altering their genetic profiles and drug sensitivities.

## Introduction

Hepatopancreatobiliary cancers include primary hepatocellular carcinoma (HCC), cholangiocarcinoma (CC), and pancreatic ductal adenocarcinoma (PDC). HCC is the fifth most common cancer worldwide, while the incidence of CC ranges from 0.44 to 1.18 cases per 100,000, and that of PDC is 7.45–7.55 cases per 100,000 [1–3]. The 5-year survival rates of PDC and CC patients are <10% and 7–20%, respectively [4, 5], whereas the HCC patients have a better prognosis with 50% 5-year survival probability [6]. Hepatopancreatobiliary tumors can be surgically resected at the early stage, which improves patient prognosis, whereas tumors at the advanced stage tend to be treated with chemotherapeutic drugs, targeted drugs, or immune checkpoint inhibitors in combination. However, it is a clinical challenge to select the appropriate drugs for the treatment of hepatopancreatobiliary cancers.

Patient-derived xenografts (PDXs) are established by directly transplanting tumor tissues from patients into immunodeficient mice, and are the most accurate in vivo model for drug screening [7]. However, the establishment of PDXs is expensive and time-consuming, which severely limits their clinical applications [8]. Patient-derived organoids (PDOs) from the tumor cells retain the pathological features [9] and drug sensitivity of the original tissues and PDX [10], and can obviate the limitations of the latter. PDOs of HCC, PDC, CC, colorectal cancer, breast cancer, gastric cancer and bladder cancer have been established so far [11–17]. Recently, we established PDOs from intrahepatic and extrahepatic CC tissues and verified the consistency between the drug sensitivity of these organoids and the actual efficacy of anticancer treatment [18, 19]. However, although most PDOs can be constructed within several weeks, those derived from the stromal cell-rich adenocarcinomas still require several months. In a recent study involving more than 20 CC patients, the longest time for establishing PDO was reported to be more than 100 weeks [20]. Given the low 5-year survival rates of patients with advanced CC or PDC, it is imperative to obtain PDOs within a reasonable time frame. We cultured PDOs in previous studies for no more than two weeks and thus only a few drugs were used in drug screening [18, 19]. Yin et al. analyzed the genetic profiles of several PDOs by mRNA sequencing and accordingly cultured them with suitable supplements [21]. Although this approach effectively shortened the duration of culture, the addition of growth factors significantly increased the costs. Cancer cells switch metabolically to anaerobic glycolysis to sustain their high proliferation rates. The end product of anaerobic glycolysis is lactate, which is used by cancer cells as a fuel [22]. Lactate supplementation promoted the proliferation of oral squamous carcinoma cells in two-dimensional culture [23], and increased the invasiveness of renal cell carcinoma cells by regulating epithelial mesenchymal transition [22]. A recent study showed that lactate enhanced the self-renewal capacity of colorectal cancer stem cells in PDOs [24]. However, it is still unclear whether lactate can promote the growth of PDOs derived from hepatopancreatobiliary tumor cells, and retain their genetic profiles and drug sensitivities. Co-cultures of peripheral blood lymphocytes with PDOs have been used for screening immune checkpoint inhibitors [25]. Therefore, it is also worth investigating whether lactate affects the interaction between PDOs and lymphocytes and the former’s sensitivity to immune checkpoint inhibitors.

The aim of this study was to evaluate the effect of lactate on the growth of PDOs derived from 2 HCC cell lines and 11 hepatopancreatobiliary tumors, and elucidate the underlying pathways. The genetic profiles and drug sensitivities of the lactate-treated PDOs were further analyzed to ascertain the feasibility of using lactate in PDO cultures.

## Results

### Lactate promoted the growth of cell line organoids in vitro

**Fig. 1:**
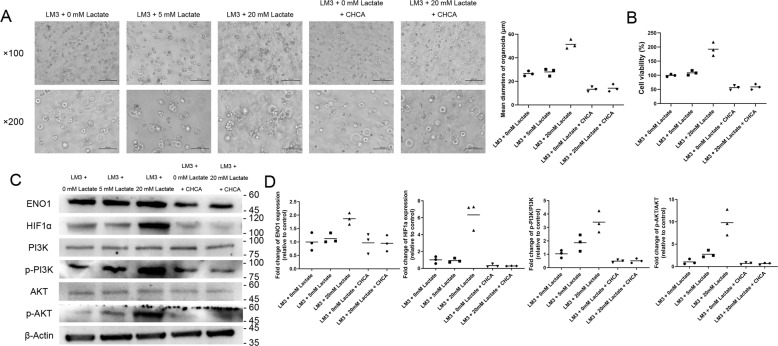
Lactate promotes the growth of LM3 organoids via the ENO1/HIF1α pathway. **A** Bright-field images of LM3 organoids treated with different concentrations of lactate or CHCA. Scale bars in ×100 row refer to 200 μm and scale bars in ×200 row refer to 100 μm. **B** Viability of LM3 organoids treated with different concentrations of lactate or CHCA. **C** Immunoblot showing bands corresponding to ENO1, HIF1α, PI3K, p-PI3K, AKT, p-AKT, and β-actin. **D** Quantification of the above proteins, p-PI3K/PI3K ratio, and the p-AKT/AKT ratio in the indicated groups. *P < 0.05.

We established organoids of two HCC cell lines (LM3 and Huh7), one PDC cell line (Panc02), and one CC cell line (RBE), and analyzed the effect of lactate on their growth. As shown in Fig. 1A, 20 mM lactate significantly increased the growth of LM3 organoids compared to the untreated controls, as indicated by the larger diameters of the lactate-treated organoids. Consistent with this, supplementation with 20 mM lactate increased the viability of the LM3 organoids by 93% (Fig. 1B), whereas lower concentrations of lactate did not have any significant effect on the growth of either (Fig. 1A, B). Similarly, 20 mM lactate significantly increased the growth of Huh7, Panc02 and RBE organoids compared to the untreated controls (Supplementary Figs. S1A, S2A and S3A). It increased the viability of the Huh7 organoids by 36%, that of the Panc02 organoids by 57%, and that of the RBE organoids by 18% (Supplementary Figs. S1B, S2B and S3B). To further confirm the pro-growth effect of lactate, the organoids were treated with the monocarboxylate transporter blocker CHCA, which inhibits the cellular intake of lactate. CHCA significantly decreased the growth of the lactate-treated organoids to even lower than that of the control group (Fig. 1A, B, Supplementary Figs. S1A, B and S2A, B, S3A, B). Taken together, lactate supplementation can significantly promote the growth of HCC organoids in vitro.

### Lactate promoted the growth of cell line organoids by activating the ENO1/HIF1α pathway

HIF1α lies downstream of lactate and ENO1 as one of the key regulators of anaerobic glycolysis. As shown in Fig. 1C, D, the ENO1 levels were significantly higher in the lactate-treated LM3 organoids compared to the control group (*P* < 0.05), and decreased nearly to that of the controls in the presence of CHCA (*P* < 0.05 vs the lactate-treated group). Likewise, HIF1α was also upregulated by 20 mM lactate (*P* < 0.05 vs control) and decreased by further addition of CHCA (p < 0.05). The PI3K/AKT pathway is downstream of HIF1α, and similar trends were observed with the expression levels of PI3K and AKT in cells treated with lactate with or without CHCA (Fig. 1C, D). Lactate supplementation increased the expression of ENO1, HIF1α, PI3K, and AKT in the Huh7 and Panc02 organoids as well (Supplementary Figs. S1C, 1D and S2C, D). However, only the expression of HIF1α was significantly increased by lactate supplementation in the RBE organoids (Supplementary Fig. S3C, D).

**Fig. 2:**
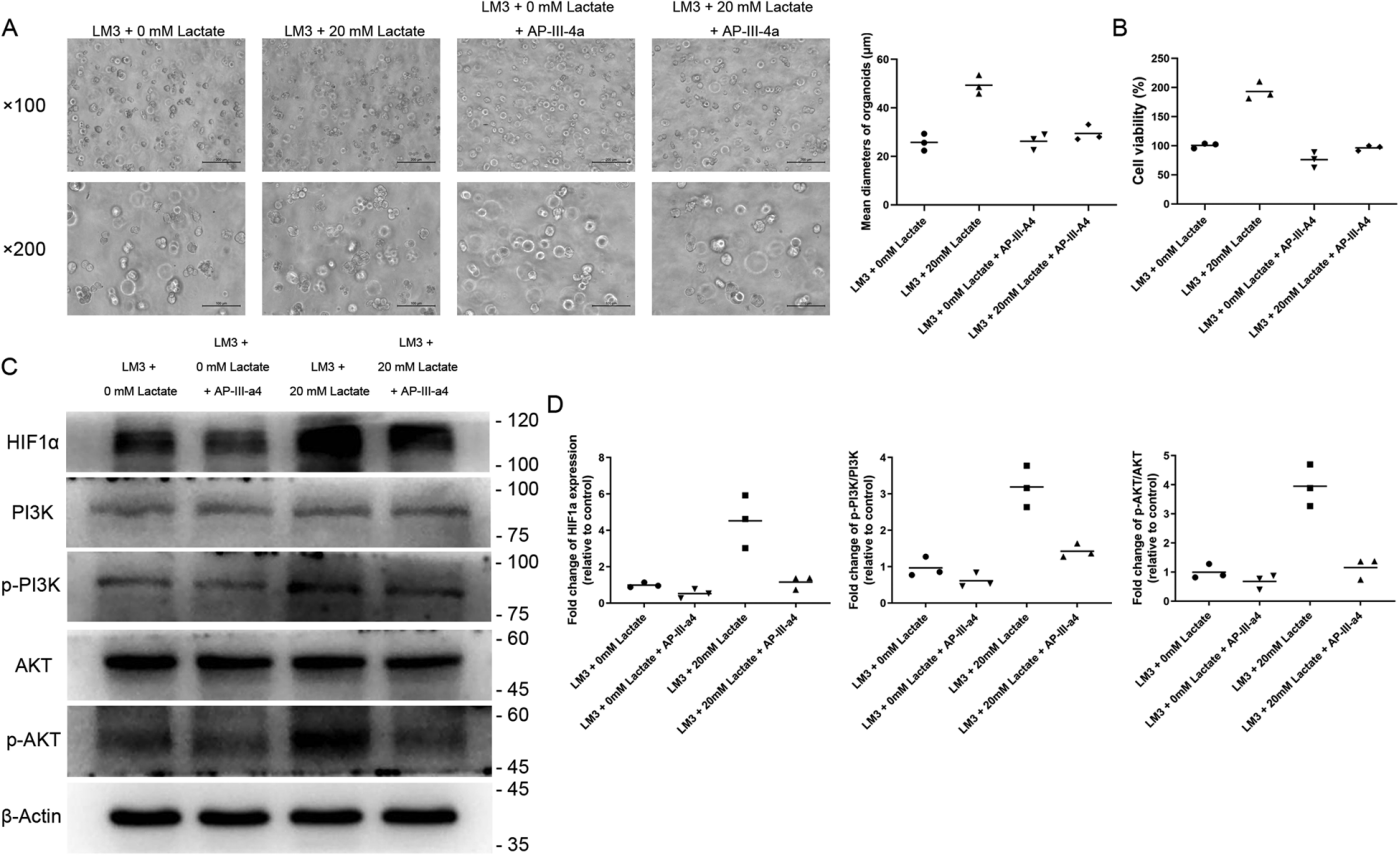
Inhibition of ENO1 reversed the effect of lactate on the growth of LM3 organoids. **A** Bright-field images of LM3 organoids treated with 20 mM lactate or AP-III-a4. Scale bars in ×100 row refer to 200 μm and scale bars in ×200 row refer to 100 μm. **B** Viability of LM3 organoids treated with 20 mM lactate or AP-III-a4. **C** Immunoblot showing bands corresponding to HIF1α, PI3K, p-PI3K, AKT, p-AKT, and β-actin. **D** Quantification of the above proteins, p-PI3K/PI3K ratio, and p-AKT/AKT ratio in the indicated groups. *P < 0.05.

To further determine whether ENO1 mediated the pro-growth effects of lactate, we treated the organoids with the pan-enolase inhibitor AP-III-a4. As shown in Fig. 2A, B, AP-III-a4 significantly inhibited the growth of the untreated and lactate-treated LM3 organoids compared to their respective controls (*P* < 0.05 for both). Furthermore, AP-III-a4 also reversed the lactate-induced increase in the expression of HIF1α, PI3K, and AKT (all *P* < 0.05), albeit not close to that of the control group. Taken together, lactate promoted the growth of the cell line-derived organoids by activating the HIFα/ENO1 pathway.

### Lactate promoted PDO growth without altering their pathological features

**Fig. 3:**
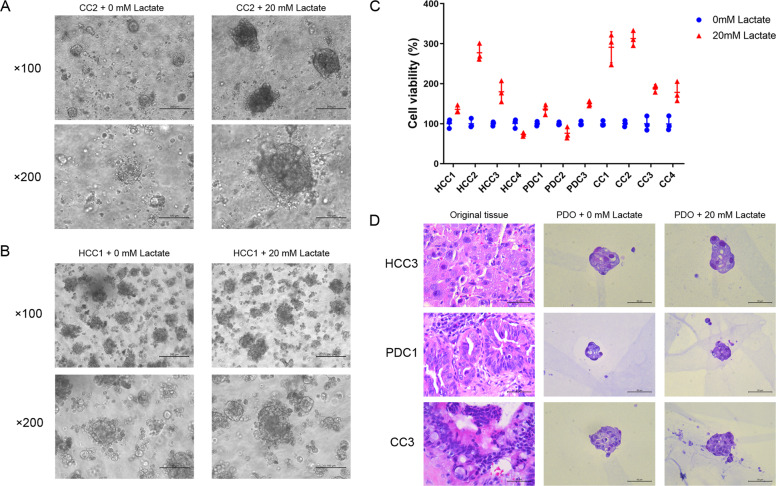
Lactate promoted the growth of PDOs from hepatopancreatobiliary tumors and preserved their pathological structure. **A** Bright-field images of CC2 PDO without or with 20 mM lactate treatment. Scale bars in ×100 row refer to 200 μm and scale bars in ×200 row refer to 100 μm. **B** Bright-field images of HCC1 PDO without or with 20 mM lactate treatment. Scale bars in ×100 row refer to 200 μm and scale bars in ×200 row refer to 100 μm. **C** Viability of 11 PDOs without or with 20 mM lactate treatment. **D** Representative images of HE-stained tumor tissues, and the HCC3, PDC1, and CC3 PDOs treated as indicated. Scale bar: 50 μm.

We established 11 PDOs from 13 tumor specimens, including 4 HCC, 3 PDC, and 4 CC PDOs. As shown in Fig. 3A, 20 mM lactate supplementation significantly increased the diameter of CC2 PDOs compared to that of the control group, but had only a slight effect on the HCC1 PDOs (Fig. 3B). In contrast, the HCC4 and PDC2 PDOs shrunk in the presence of lactate (data not shown). Furthermore, 20 mM lactate increased the viability of 9 PDOs by 135% to 317%, and decreased that of 2 PDOs by 25–27% (Fig. 3C). As shown in Fig. 3D, lactate supplementation did not alter the histopathological features or the structure of the PDOs, which retained the characteristics of the parent tissues. Immunohistochemical staining further compared the protein expression in PDOs and parent tumor tissues. The results indicated that AFP expressions and glycogen distributions were similar in parent tumor tissue and HCC PDOs with or without lactate supplementation (Supplementary Fig. S4). Besides, lactate supplementation did not alter the expressions of CK7 and MUC1 in PDC organoid and CC organoid (Supplementary Fig. S4).

### Lactate treatment conserved the genetic profile of the PDOs

**Fig. 4:**
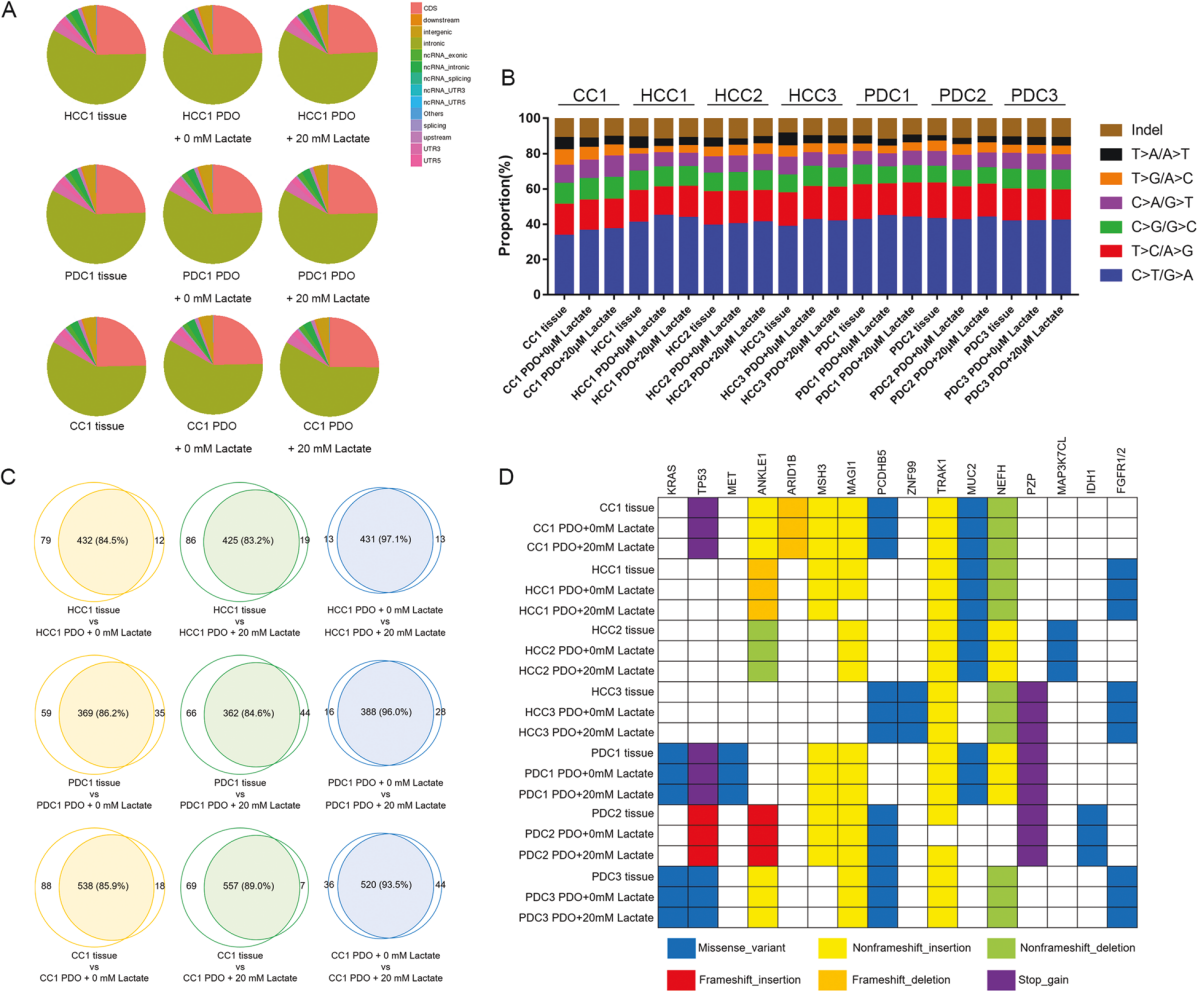
Lactate maintained the genetic profiles of PDOs. **A** The number of SNPs in the different regions of the genome in original tissues and PDOs without or with 20 mM lactate treatment. The regions are shown in the legends. **B** The proportion of the six types of base substitutions and InDels in original tissues and PDOs without or with 20 mM lactate treatment. **C** The similarity of genetic profiles between original tissues and PDOs without or with 20 mM lactate treatment. **D** Representative genetic variants in original tissues and PDOs without or with 20 mM lactate treatment. The types of variants are shown in the legends.

The genetic profiles of seven PDOs, including three HCC, three PDC, and one CC PDO, were determined by WES. The proportion of SNPs in the different regions of the genome in all seven pairs of parent tumor tissues and PDOs are shown in Fig. 4A and Supplementary Fig. S5A, which indicates no significant difference between the tumor tissues and PDOs regardless of lactate treatment. The distribution of base substitutions and InDels is shown in Fig. 4B. The C > T/G > A transversion was the most common base substitution, followed by T > C/A > G. There were only a few differences in the number of base substitutions and InDels between PDOs and parent tissues, and lactate treatment also did not have a significant effect. Furthermore, the parent tissues had 83% to 95.7% similar somatic SNVs and InDels with the untreated PDOs, whereas the similarity with lactate-treated PDOs ranged from 83.2 to 94.1% (Fig. 4C and Supplementary Fig. S5B). In addition, the similarity between the untreated and lactate-treated PDOs was at least 93.2%. Some representative genetic variants are shown in Fig. 4D. Variants of *MAGI1*, *TRAK1*, and *NEFH* were most common, whereas only PDCs and CC harbored variants of *KRAS*, *TP53*, and *MET*. Three cancers harbored *FGFR1/2* missense variant and only one cancer harbored *IDH1* missense variant. In general, genetic variants were consistent between the parent tissues and PDOs regardless of lactate treatment, indicating that lactate did not affect the genetic profiles of the PDOs.

### Lactate did not alter the drug sensitivity of PDOs

**Fig. 5:**
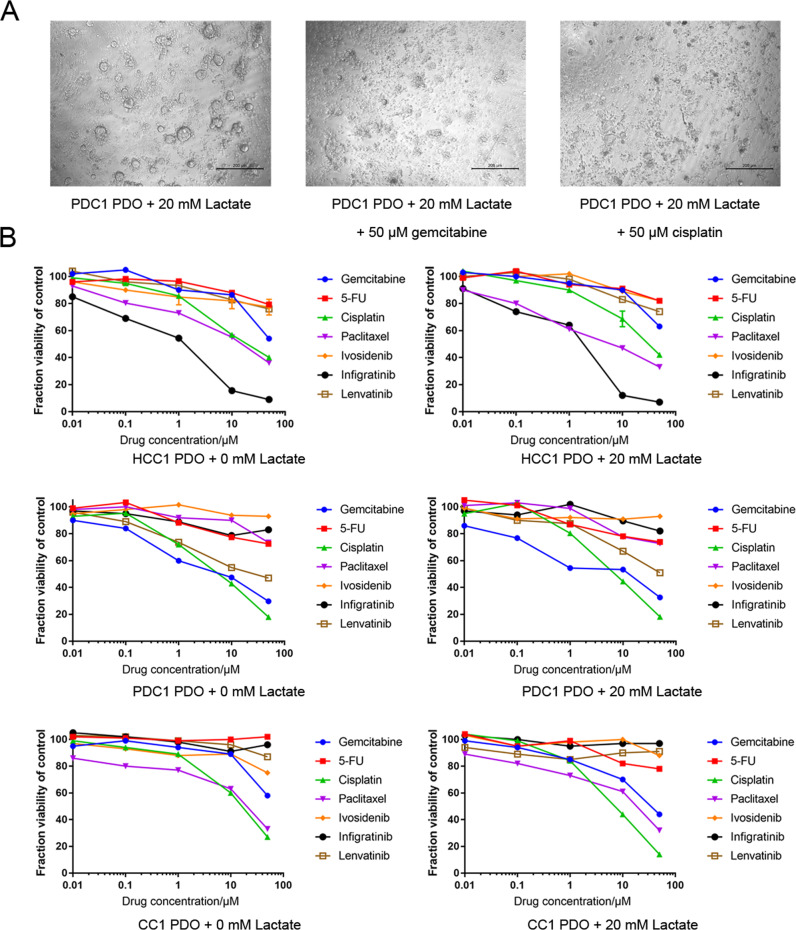
Lactate maintained the drug sensitivity of PDOs. **A** Representative images of the PDC1 PDOs treated with vehicle, 50 μM gemcitabine, and 50 μM cisplatin. **B** Dose–response curves of HCC1, PDC1, and CC1 PDOs without or with 20 mM lactate treatment to 7 drugs (indicated in the legends).

**Fig. 6:**
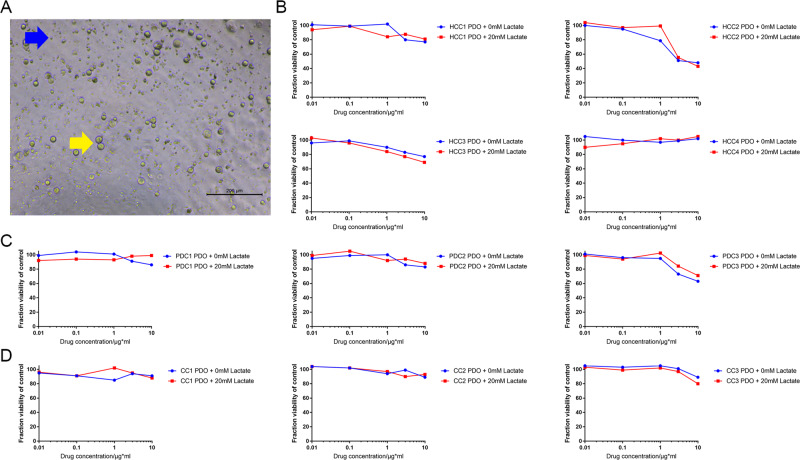
Lactate retained the sensitivity of the PDOs to immune checkpoint inhibitors. **A** Representative images of co-cultured CC2 PDO and PBMCs. Blue arrow indicates PMBC and yellow arrow indicates CC2 PDO. **B** Dose–response curves of ten PDOs of four HCCs without or with 20 mM lactate treatment to tislelizumab. **C** Dose–response curves of ten PDOs of three PDCs without or with 20 mM lactate treatment to tislelizumab. **D** Dose–response curves of ten PDOs of three CCs without or with 20 mM lactate treatment to tislelizumab.

The different PDOs were recovered in the absence of lactate for 2 days, and exposed to chemotherapeutic drugs with or without lactate. As shown in the bright-field images in Fig. 5A, the PDC1 PDOs treated with 50 μM gemcitabine and 50 μM cisplatin were significantly smaller, and showed extensive apoptosis compared to the untreated cells, regardless of lactate supplementation. The dose–response curves for cell viability were consistent with the images (Fig. 5B). The results of drug screening for ten pairs of PDOs without and with 20 mM lactate treatment are shown in Fig. 5B and Supplementary Fig. S6. Although lactate supplementation had a minor effect on the drug response curves in case of resistant cells, the sensitivity of the PDOs to specific drugs was unaffected. All IC_50_ of seven therapeutic drugs in PDOs with or without lactate supplementation were listed in Table 1. At present, drugs are usually defined as sensitive or resistant according to whether the IC_50_ is lower than 10 μM. Some HCC PDOs had certain sensitivity to traditional chemotherapy drugs such as gemcitabine and cisplatin. HCC has high sensitivity to infigratinib, and three of the four HCC PDOs had an IC_50_ of lower than 10 μM in this study. The drug resistance rate of PDC and CC was relatively high, and only one third of PDOs were sensitive to gemcitabine and infigratinib. Significant changes in IC_50_ of some drugs were observed only in three PDOs after the treatment of lactate. For example, the IC_50_ of paclitaxel on HCC1 PDO changed from 14.72 to 8.16 μM after the treatment of lactate, the IC_50_ of lenvatinib on HCC1 PDO changed from 9.07 to 14.73 μM, and the IC_50_ of cisplatin on HCC1 PDO changed from 11.89 to 9.29 μM. We also co-cultured patient-derived PBMCs with PDOs to assess tislelizumab sensitivity (Fig. 6A). As shown in Fig. 6B–D, 20 mM lactate did not alter the sensitivities of four HCC PDOs, three PDC PDOs, and three CC PDOs to tislelizumab. In addition, PDC3 was the most sensitive to tislelizumab, and showed a 60–70% decrease in viability in response to 10 μg/mL of the drug.

**Table 1:** IC_50_ of seven therapeutic drugs in PDOs with or without lactate.

	Gemcitabine	5-Fluorouracil	Cisplatin	Paclitaxel	Ivosidenib	Infigratinib	Lenvatinib
HCC1 PDO	80.6 μM	>100 μM	14.52 μM	***14.72*** ***μM***	>100 μM	0.81 μM	>100 μM
HCC1 PDO + lactate	>100 μM	>100 μM	33.32 μM	***8.16*** ***μM***	>100 μM	2.22 μM	>100 μM
HCC2 PDO	6.77 μM	>100 μM	38.83 μM	31.86 μM	>100 μM	>100 μM	10.51 μM
HCC2 PDO + lactate	8.19 μM	>100 μM	16.66 μM	27.69 μM	>100 μM	>100 μM	12.9 μM
HCC3 PDO	63.82 μM	>100 μM	2.73 μM	>100 μM	>100 μM	4.18 μM	>100 μM
HCC3 + PDO + lactate	>100 μM	>100 μM	5.17 μM	>100 μM	>100 μM	2.99 μM	>100 μM
HCC4 PDO	14.90 μM	>100 μM	6.26 μM	26.73 μM	>100 μM	5.35 μM	***9.07*** ***μM***
HCC4 + PDO + lactate	20.56 μM	>100 μM	8.33 μM	45.17 μM	>100 μM	7.10 μM	***14.73*** ***μM***
PC1 PDO	4.55 μM	>100 μM	7.05 μM	>100 μM	>100 μM	>100 μM	14.93 μM
PC1 PDO + lactate	2.88 μM	>100 μM	7.84 μM	>100 μM	>100 μM	>100 μM	42.37 μM
PC2 PDO	> 100 μM	>100 μM	>100 μM	38.7 μM	7.11 μM	>100 μM	>100 μM
PC2 PDO + lactate	>100 μM	>100 μM	>100 μM	44.67 μM	5.37 μM	>100 μM	>100 μM
PC3 PDO	> 100 μM	>100 μM	>100 μM	>100 μM	>100 μM	5.54 μM	>100 μM
PC3 PDO + lactate	>100 μM	>100 μM	>100 μM	>100 μM	>100 μM	2.33 μM	>100 μM
CC1 PDO	91.93 μM	>100 μM	***11.89*** ***μM***	19.8 μM	>100 μM	>100 μM	>100 μM
CC1 PDO + lactate	25.4 μM	>100 μM	***9.29*** ***μM***	22.59 μM	>100 μM	>100 μM	>100 μM
CC2 PDO	> 100 μM	>100 μM	>100 μM	37.31 μM	>100 μM	>100 μM	>100 μM
CC2 PDO + lactate	>100 μM	>100 μM	>100 μM	45.84 μM	>100 μM	>100 μM	>100 μM
CC3 PDO	4.17 μM	>100 μM	40.74 μM	25.1 μM	>100 μM	3.07 μM	78.31 μM
CC3 PDO + lactate	7.82 μM	>100 μM	31.35 μM	31.66 μM	>100 μM	6.04 μM	>100 μM

*IC*_*50*_ 50% inhibitory concentration, *HCC* hepatocellular carcinoma, *PDC* pancreatic ductal adenocarcinoma, *CC* cholangiocarcinoma, *PDO* patient-derived organoid.

Bold and italic values represent totally different drug sensitivities between PDOs with or without lactate.

## Discussion

This study is the first to evaluate the impact of lactate supplementation on the growth, tumorigenicity and drug sensitivity of PDOs derived from hepatopancreatobiliary tumors. Our findings indicate that high concentration of lactate can promote the growth of the PDOs without affecting their genetic profiles and drug sensitivity.

To explore the mechanistic basis of the pro-growth effects of lactate, we established organoids from two HCC cell lines, one PDC cell line and one RBE cell line, since the amount of protein extracted from PDOs is insufficient for routine molecular analyses. We found that the oncogene HIF1α was significantly upregulated in the organoids treated with 20 mM lactate. HIF1α is activated by the high levels of reactive oxygen species, succinate, and lactate in a hypoxic environment [26]. The PI3K/AKT pathway lies downstream of HIF1α and can activate HIF1α through a positive feedback loop [27]. Enolase is one of the key enzymes in anaerobic glycolysis, which catalyzes the conversion of 2-phosphoglycerate to phosphoenolpyruvate. Studies show that enolase regulates the production of lactate in normal cells [28, 29]. In a previous study, we found that serum ENO1 is significantly elevated in HCC patients with microvascular invasion (MVI) compared to those without MVI [30]. Principe et al. also reported that high expression of ENO1 promoted the proliferation and metastasis of pancreatic cancer cells [31]. Given the role of ENO1 in anaerobic glycolysis and the metabolic switch in cancer cells, we hypothesized that it likely mediates the effects of lactate on the growth of cell line-derived organoids. Indeed, lactate supplementation also increased the expression of ENO1 in the organoids, which was suggestive of distinct glucose metabolism profiles of cancer and non-cancer cells. Furthermore, ENO1 promotes the proliferation and metastasis of breast cancer cells through the PI3K/AKT pathway [32], and in turn is upregulated by HIF1α [33, 34]. Consistent with this, the pan-enolase inhibitor AP-III-a4 significantly decreased the expression levels of PI3K, AKT, and HIF1α in the lactate-treated organoids. This also suggested that ENO1 may regulate the expression of HIF1α in hepatopancreatobiliary cancers. Nevertheless, inhibition of ENO1 could not completely attenuate HIF1α and PI3K/AKT pathways compared to the control group, indicating the involvement of other pathways. Several chemotherapeutic drugs target metabolic pathways in cancer cells, including glycolysis, mitochondrial respiration and glutaminolysis [35]. Taken together, lactate promoted the growth of organoids via the HIF1α/PI3K/AKT pathway and partly via the ENO1 pathway, which could be potential targets for hepatopancreatobiliary cancer treatment.

The concentration of lactate is a determinant of the growth rate of organoids. A previous study showed that 5 mM lactate promoted the formation of organoids from cells derived from oral or colorectal tumors [24, 36]. However, we found that concentrations lower than 20 mM had minimal effect on the growth of cell line organoids, and 5 mM lactate only slightly activated HIF1α/PI3K/AKT and had no effect on the expression of ENO1. Furthermore, 20 mM lactate promoted the growth of LM3 organoids by 93%, that of Huh7 organoids by 36%, that of Panc02 organoids by 57% and that of RBE organoids by 18%, indicating that the cell type is also a factor in lactate response. Likewise, lactate increased the growth of 9/11 PDOs by 35% to 217%, and the growth rate was higher for the cholangiosarcoma-derived organoids, indicating that lactate may be more important for the growth of these tumors. However, this hypothesis could not be explored further due to the small sample size. Conversely, two PDOs from PDC were inhibited by lactate, which can be attributed to the higher baseline growth rate of both PDOs, resulting in greater lactate production. Therefore, lactate supplementation further lowered the pH of the medium below the suitable threshold, which inhibited the growth of PDOs. Nevertheless, lactate had no significant effect on the pathological structure or the genetic profiles of any of the PDOs. According to WES results, the minimum similarity between the untreated and lactate-treated PDOs was 93.2%. Furthermore, the similarities between lactate-treated PDOs and the parent tissues ranged from 83.2 to 94.1%. Lee et al. reported that 11 of 15 PDOs from bladder tumors were >80% genetically similar to the corresponding parent tissues [17]. Broutier et al. also reported that approximately 84% of the cancer-related somatic variants in parent tissues were retained in the corresponding PDOs from liver tumors [12]. Both groups proposed that >80% genetic similarity between PDOs and corresponding parent tissues indicated appropriate PDO culture, and also confirmed the effects of lactate.

We tested the impact of lactate on the response to gemcitabine, 5-fluorouracil, cisplatin, paclitaxel, and three targeted drugs including ivosidenib, infigratinib, and Lenvatinib. The four chemotherapeutic drugs are used as the first-line treatment against PDC and CC, and lenvatinib is the first-line targeted drug for HCC. Ivosidenib is an inhibitor of isocitrate dehydrogenase 1 (IDH1) and significantly improved the progression-free survival of IDH1-mutant CC patients compared to that of the placebo arm [37]. Infigratinib is an inhibitor of fibroblast growth factor receptor 2 (FGFR2) and has shown promising therapeutic effects in previously treated FGFR2-mutant cholangiocarcinoma patients [38]. Furthermore, infigratinib alone or in combination with other drugs is also effective in FGFR-mutant HCC patients [39]. Our results suggested that traditional chemotherapy drugs such as gemcitabine and cisplatin may also have significant effects on HCC. Besides, some hepatopancreatobiliary cancers were found to be highly sensitive to infiratinib, which may be further used in clinical treatment in the future.

Previous reports indicate that lactate may enhance the resistance of cancer cells to anticancer drugs and radiotherapy [40, 41]. Therefore, all PDOs were allowed to recover in lactate-free medium before drug screening. Lactate treatment only had a little effect on the drug sensitivities of the PDOs. The IC_50_ of paclitaxel, lenvatinib, and cisplatin changed significantly in HCC1 PDO, HCC4 PDO, and CC1 PDO with lactate treatment, respectively. This suggested that there was still some space for optimization of lactate treatment. The sensitivity of the PDOs to ivosidenib or infigratinib was consistent with the IDH1 or FGFR1/2 variants in genetic profiles.

Immune checkpoint inhibitors have been widely used for treating hepatopancreatobiliary cancers in recent years. However, the sensitivity of PDOs to immune checkpoint inhibitors has not been assessed so far due to the lack of immune cells in the culture medium. Therefore, we co-cultured the PDOs with patient-derived PBMCs, and tested their sensitivity to the anti-PD1 antibody tislelizumab. Lactate also did not affect the sensitivity to tislelizumab, and the HCC2, HCC3, and PDC3 PDOs were particularly sensitive. Although lactate did not significantly affect the interaction between PDOs and lymphocytes, the dose–response curves were still influenced by the viability of PMBCs. Therefore, the sensitivity of PDOs to tislelizumab should be further analyzed in combination with actual clinical effects and drug use.

There are some limitations in this study that ought to be considered. We only used two HCC cell lines, one PDC cell line and one CC cell line to explore the mechanistic basis of lactate, we cannot exclude the possibility of the involvement of other cancer-specific pathways. Second, we used AP-III-a4 to inhibit ENO1 due to the relatively long duration of siRNA experiments. However, gene silencing would specifically inhibit the expression and function of ENO1 rather than all enolases. Third, few patients who provided the tissue specimens received preoperative chemotherapy, targeted therapy, or combination therapy. Therefore, the results of drug screening cannot be verified by the actual therapeutic effects and can only be evaluated after long-term follow-up to obtain the 3-year or 5-year survival rates. Fourth, investigating mRNA expression profiles would be useful to further compare the differences between PDOs and primary tissues. Unfortunately, the remaining PDOs are not enough for mRNA expression detection. Finally, the DNA samples of four PDO samples were degraded due to poor preservation, leading to incomplete WES results.

In conclusion, 20 mM lactate promoted the growth of most PDOs from hepatopancreatobiliary tumors via the ENO1/HIF1α pathway without affecting their genetic profiles and drug sensitivity. Therefore, lactate supplementation should be considered for expanding cancer cells in vitro.

## Materials and methods

### Cell line culture

The cell lines LM3 (XafhBio), Huh7, Panc02, and RBE (National Collection of Authenticated Cell Cultures) were authenticated and cultured in Dulbecco’s modified Eagle’s medium (DMEM; GIBCO, CA, USA) supplemented with 10% fetal bovine serum (FBS; GIBCO, CA, USA) and 1% penicillin/streptomycin (ThermoFisher, MA, USA) at 37 °C under 5% CO_2_. After three passages, ~2–5 × 10^5^ LM3 or Huh7 cells were resuspended in a 1:1 mixture of growth factor-reduced Matrigel matrix (Corning, NY, USA) and DMEM, and seeded onto six-well plates pre-coated with the same mixture. Each well was filled with 2 mL complete DMEM supplemented with different concentrations of L-lactate (1, 5, 10, and 20 mM), 1 mM α-cyano-4-hydroxycinnamic acid (CHCA) or 1 μM AP-III-a4. The cells were cultured of 7 days, and the organoids were observed microscopically. The mean diameter of the organoids was measured in three high-power fields. The viability of the cells was determined using the CellTiter-Glo 3D Cell-Viability kit (Promega, WI, USA).

### Western blotting

Total protein was extracted from LM3 or Huh7 organoids on ice using RIPA lysis buffer supplemented with protease inhibitor and phosphatase inhibitors. The protein content was measured, and 20 mg of protein per sample was resolved by SDS-PAGE and transferred to a PVDF membrane. After blocking with 5% bovine serum albumin, the membrane was incubated overnight with primary antibodies against α-enolase (ENO1, Abcam, Cambridge, UK), hypoxia-inducible factor-1α (HIF1α, Abcam, Cambridge, UK), AKT (Abcam, Cambridge, UK), p-AKT (Abcam, Cambridge, UK), PI3 kinase (PI3K, Abcam, Cambridge, UK), p-PI3K (Cell Signaling Technology, MA, USA), and β-actin (Beyotime, China). Following incubation with the suitable secondary antibodies, the protein bands were developed using BeyoECL Plus kit (Beyotime, China). The signal intensities of the bands were measured using Image-pro plus 6.0 (Media Cybernetics, TX, USA), and converted to fold change between the control and experiment groups. Each experiment was performed in triplicate.

### Culture of PDO

A total of 13 hepatopancreatobiliary tumor samples, including 5 HCC, 4 PDC, and 4 CC specimens, were collected during surgical resection and transferred on ice within 4 h. The tissues were washed thrice with sterile saline, minced into 0.5–1 mm^3^ pieces, and digested with 2 mg/mL collagenase D (Roche, Basel, Switzerland) for 2–3 h at 37 °C under 5% CO_2_. The mixture was agitated lightly once every 30 min. Then dissociated single cells or clusters were passed through a 100-µm cell strainer and centrifuged at 300×*g* for 5 min at room temperature. Following erythrocyte lysis, the number of cancer cells were counted and resuspended at the density of 5 × 10^5^ cells/mL in complete DMEM/F12. Half of the PDOs of each type were resuspended in medium supplemented with 20 mM l-lactate immediately after digestion. The wells of a 24-well plate were each coated with 200 μL 1:1 mixture of growth factor-reduced Matrigel and complete DMEM, and 200 μL of the cell suspension was seeded onto each pre-coated well along with 500 µL complete DMEM/F12. The culture medium was replaced every 3–4 days and the PDOs were grown for 2 weeks. The viability of the PDOs was evaluated using the CellTiter-Glo 3D Cell-Viability assay.

The PDOs were cultured in advanced DMEM/F12 (Gibco, CS, USA) supplemented with 1x penicillin/streptomycin (ThermoFisher, MA, USA), 1× Glutamax (ThermoFisher, MA, USA), 10 mM HEPES (ThermoFisher, MA, USA), 1× B27 supplement (Gibco, CS, USA), 1× N2 supplement (Gibco, CS, USA), 10 nM gastrin (Sigma, MO, USA), 5 μM A83-01 (Tocris, Bristol, UK), 10 µM Y-27632 (Tocris, Bristol, UK), 50 ng/mL recombinant human epidermal growth factor (EGF, PeproTech, NJ, USA), 100 ng/mL recombinant human fibroblast growth factor 10 (FGF10, PeproTech, NJ, USA), 500 ng/mL recombinant human R-Spondin1 (R-Spo1, PeproTech, NJ, USA) and 10% v/v Afamin/Wnt3a CM (MBL Life Science, Japan) [12]. The HCC-derived PDOs additionally required 10 μM forskolin (Tocris, Bristol, UK) and 25 ng/mL recombinant human hepatocyte growth factor (HGF, PeproTech, NJ, USA), whereas 25 ng/mL recombinant human Noggin (PeproTech, NJ, USA) was added for the CC and PDC organoids [11, 13].

The tissue samples were retrieved following approval from the ethics committee of the Second Affiliated Hospital, Zhejiang University, School of Medicine (No. 2019-408). The study was performed in compliance with the principles of the Declaration of Helsinki (2013), and all patients provided written informed consent prior to surgery.

### Histological staining

The PDOs were digested with 1.5 mg/mL dispase II (Roche, Basel, Switzerland) and centrifuged at 300×*g* for 5 min. Some of the pellets were fixed in 4% paraformaldehyde and resuspended evenly in 4% low-melting agarose after centrifugation. The agarose blocks and tumor specimens were embedded into paraffin blocks after dehydration and cut into 4-μm thick sections. Hematoxylin and eosin (H&E) staining was performed as per standard protocols. Immunohistochemical staining was performed according to standard protocols and the following antibodies were used: anti-alpha fetoprotein (AFP; Abcam, Cambridge, UK, 1:200), cytokeratin-7 (CK7; Abcam, Cambridge, UK, 1:2000), and mucin-1 (MUC1; Abcam, Cambridge, UK, 1:500). Periodic acid-Schiff (PAS) staining was performed to assess the distribution of glycogen in original cancer specimens as well as PDOs.

### Whole-exome sequencing (WES)

Total DNA was extracted from the tumor tissues and PDOs using GenElute Mammalian Genomic DNA miniprep kit (Sigma, MO, USA) according to the manufacturer’s instructions. WES was performed on the Illumina platform as described in previous studies [18]. SAMtools were used for detecting single nucleotide polymorphisms (SNPs), and insertions and deletions (InDels). Copy number variations (CNVs) were identified using Control-FREEC. The somatic single nucleotide variations (SNVs), InDels, and CNVs were detected using muTect, Strelka, and Control-FREEC respectively.

### Drug screening

The PDOs were dissociated into single cells by incubating with 0.25% trypsin-EDTA for 5–10 minutes. The single cells were resuspended in complete DMEM containing 2% Matrigel, and 100–200 cells/22.5 μL were seeded into each well of a pre-coated 384-well plate. After culturing for two days, 2.5 μL complete DMEM supplemented with different concentrations (0.1, 1, 10, 100, or 500 µM) of gemcitabine, 5-fluorouracil, cisplatin, paclitaxel, ivosidenib, infigratinib, or Lenvatinib was added. The viability of the cells was evaluated 96 h later using the CellTiter-Glo 3D Cell-Viability kit. Negative controls were included and each experiment was performed in triplicate.

In addition, 2 mL peripheral blood was also collected from the patients, and peripheral blood mononuclear cells (PBMCs) were isolated by Ficoll-Paque density-gradient separation. Following RBC lysis, the PBMCs were cultured in RPMI 1640 medium supplemented with 1× penicillin/streptomycin, 1× Glutamax, and 25 ng/mL recombinant human IL-2 (PeproTech, NJ, USA) for 1 week. The PBMCs were counted and co-cultured with PDOs at the ratio of 50–100:1 in the presence of tislelizumab.

### Statistical analysis

All statistical analyses, the figures and 50% inhibitory concentration (IC_50_) were generated using GraphPad Prism software 7.0 (GraphPad Software, CA, USA). Mann–Whitney tests were used for the comparisons between two groups in each figure, due to the data in each figure may not meet the assumptions of *t* tests.

## Supplementary information

Supplementary figure legends

Figure S1 Lactate promoted the growth of Huh7 organoids via ENO1/HIF1α pathway.

Figure S2 Lactate promoted the growth of Panc02 organoids via ENO1/HIF1α pathway.

Figure S3 Lactate promoted the growth of RBE organoids via HIF1α pathway.

Figure S4 Immunohistochemical staining and PAS staining of parent cancer tissues and cancer PDOs with or without lactate supplementation.

Figure S5 Lactate retained the genetic profiles of PDOs from hepatopancreatobiliary cancers.

Figure S6 Dose-response curves of HCC2, HCC3, HCC4, PDC2, PDC3, CC2 and CC3 PDOs without or with 20 mM lactate treatment to 7 drugs

Original western blot

## Data Availability

The datasets used and analyzed during the current study are available from the corresponding author on reasonable request. The WES data in the current study has been uploaded in the NCBI database and it can be available from the corresponding author on reasonable request.
