# Supplementary material for: Lactate promotes the growth of patient-derived organoids from hepatopancreatobiliary cancers via ENO1/HIF1α pathway and does not affect their drug sensitivities
Source: Cell Death Discov. 2022 Apr 20;8:214. doi: 10.1038/s41420-022-01014-4 (PMC9021221; doi:10.1038/s41420-022-01014-4)
Supplement: Supplementary file 1 — Supplementary figure legends [file 41420_2022_1014_MOESM1_ESM.docx]

**Supplementary figure legends**

Figure S1 Lactate promoted the growth of Huh7 organoids via ENO1/HIF1α pathway. (A) Bright field images of Huh7 organoids treated with different concentrations of lactate or CHCA. Scale bars in ×100 row refer to 200 μm and scale bars in ×200 row refer to 100 μm. (B) Viability of Huh7 organoids treated with different concentrations of lactate or CHCA. (C) Immunoblot showing bands corresponding to ENO1, HIF1α, PI3K, p-PI3K, AKT, p-AKT and β-actin. (D) Quantification of the above proteins, p-PI3K/PI3K ratio and p-AKT/AKT ratio. *: p<0.05.

Figure S2 Lactate promoted the growth of Panc02 organoids via ENO1/HIF1α pathway. (A) Bright field images of Panc02 organoids treated with different concentrations of lactate or CHCA. Scale bars in ×100 row refer to 200 μm and scale bars in ×200 row refer to 100 μm. (B) Viability of Panc02 organoids treated with different concentrations of lactate or CHCA. (C) Immunoblot showing bands corresponding to ENO1, HIF1α, PI3K, p-PI3K, AKT, p-AKT and β-actin. (D) Quantification of the above proteins, p-PI3K/PI3K ratio and p-AKT/AKT ratio. *: p<0.05.

Figure S3 Lactate promoted the growth of RBE organoids via HIF1α pathway. (A) Bright field images of RBE organoids treated with different concentrations of lactate or CHCA. Scale bars in ×100 row refer to 200 μm and scale bars in ×200 row refer to 100 μm. (B) Viability of RBE organoids treated with different concentrations of lactate or CHCA. (C) Immunoblot showing bands corresponding to ENO1, HIF1α, PI3K, p-PI3K, AKT, p-AKT and β-actin. (D) Quantification of the above proteins, p-PI3K/PI3K ratio and p-AKT/AKT ratio. *: p<0.05.

Figure S4 Immunohistochemical staining and PAS staining of parent cancer tissues and cancer PDOs with or without lactate supplementation. The antigens used in immunohistochemical staining include AFP, CK7 and MUC1. Scale bar: 50 μm

Figure S5 Lactate retained the genetic profiles of PDOs from hepatopancreatobiliary cancers. (A) The number of SNPs in the different regions of the genome in original tissues and PDOs without or with 20 mM lactate. The regions are shown in the legends. (B) The similarity of genetic profiles between original tissues and PDOs without or with 20 mM lactate.

Figure S6 Dose-response curves of HCC2, HCC3, HCC4, PDC2, PDC3, CC2 and CC3 PDOs without or with 20 mM lactate treatment to 7 drugs (indicated in the legends).
