# Supplementary figures and images for: Lactate promotes the growth of patient-derived organoids from hepatopancreatobiliary cancers via ENO1/HIF1α pathway and does not affect their drug sensitivities
Source: Cell Death Discov. 2022 Apr 20;8:214. doi: 10.1038/s41420-022-01014-4 (PMC9021221; doi:10.1038/s41420-022-01014-4)

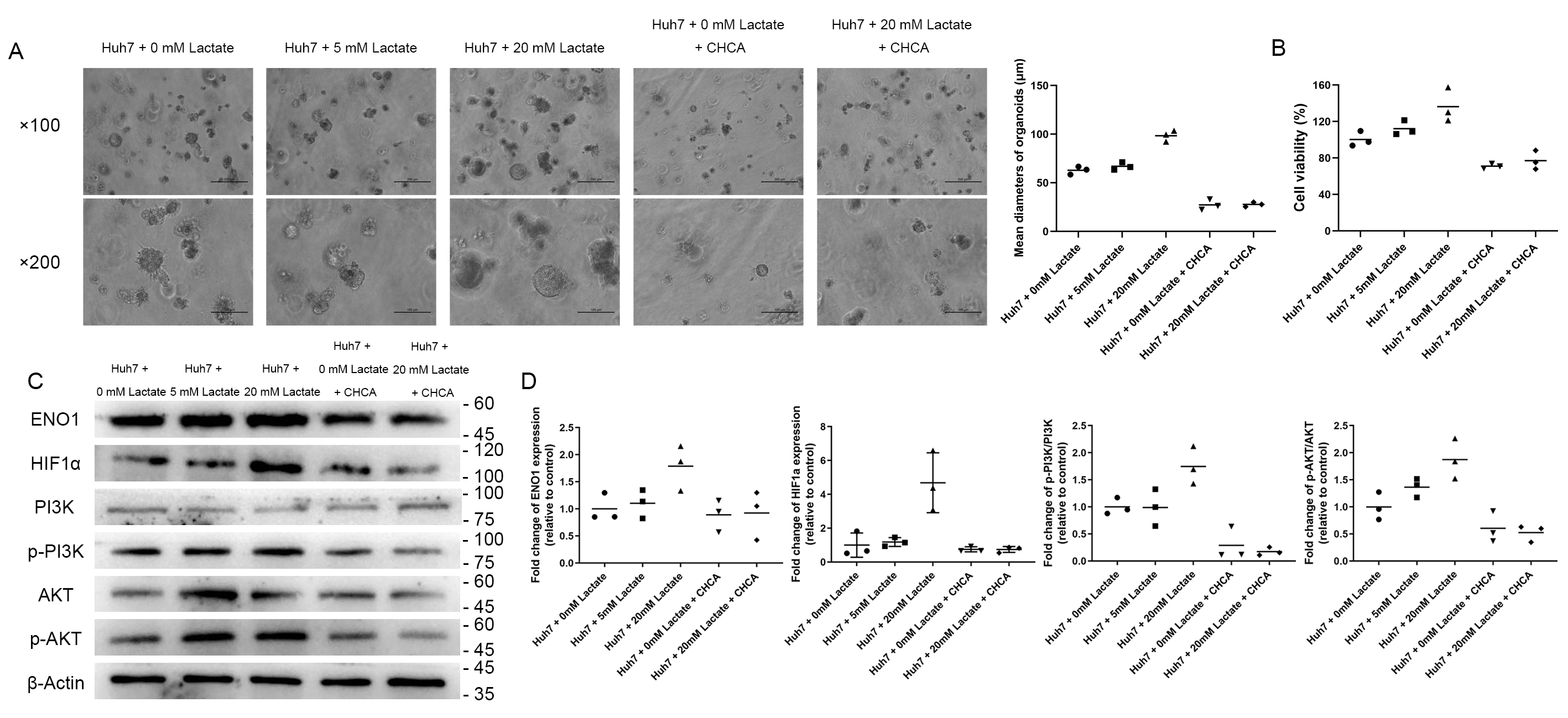

Supplement: Supplementary file 2 — Figure S1 Lactate promoted the growth of Huh7 organoids via ENO1/HIF1α pathway. [file 41420_2022_1014_MOESM2_ESM.tif]

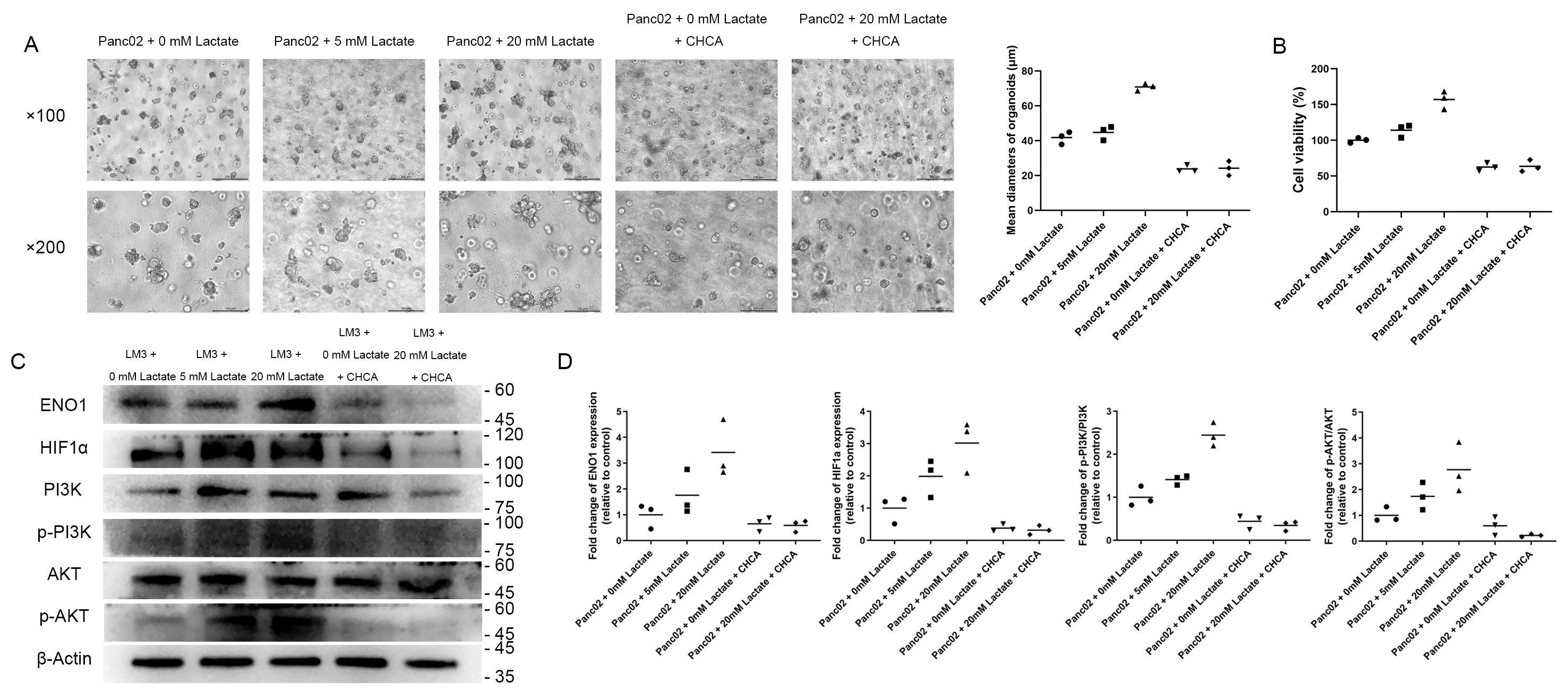

Supplement: Supplementary file 3 — Figure S2 Lactate promoted the growth of Panc02 organoids via ENO1/HIF1α pathway. [file 41420_2022_1014_MOESM3_ESM.tif]

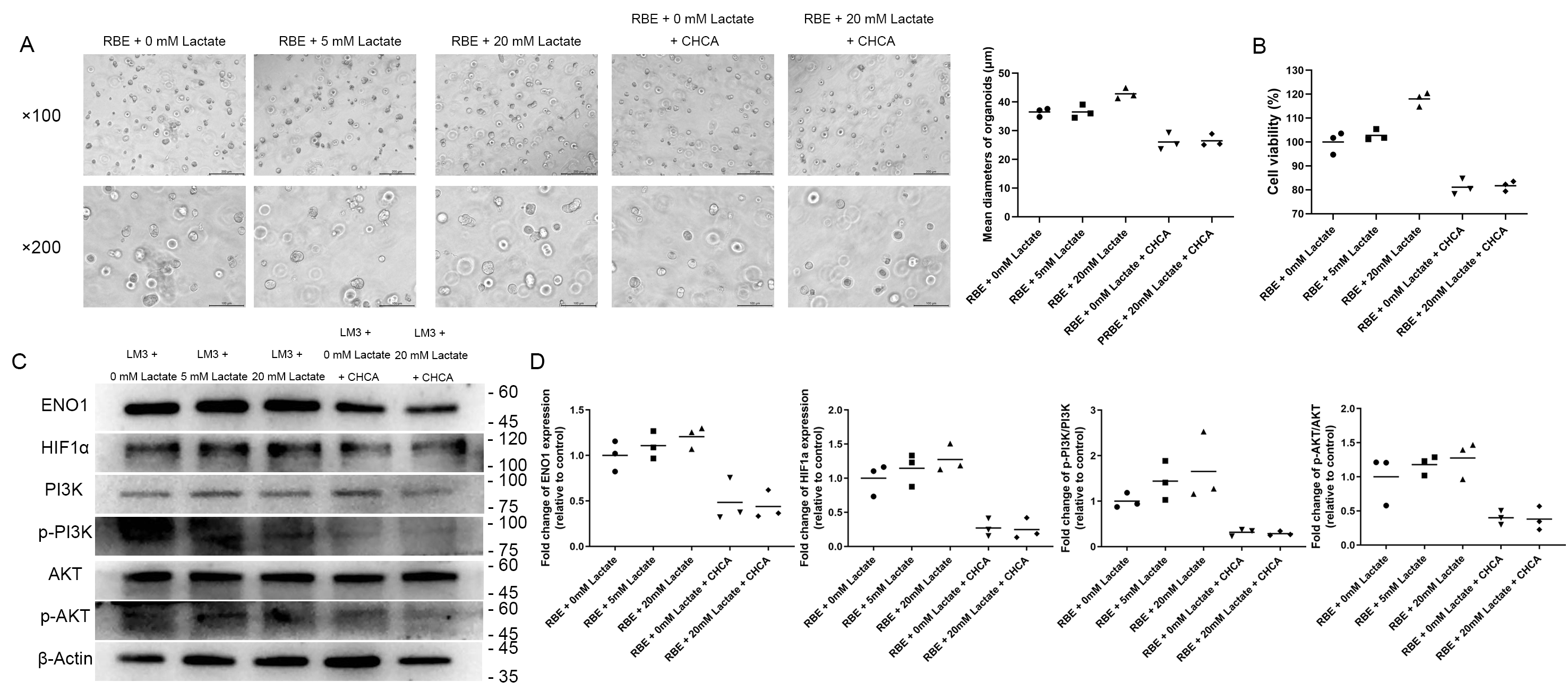

Supplement: Supplementary file 4 — Figure S3 Lactate promoted the growth of RBE organoids via HIF1α pathway. [file 41420_2022_1014_MOESM4_ESM.tif]

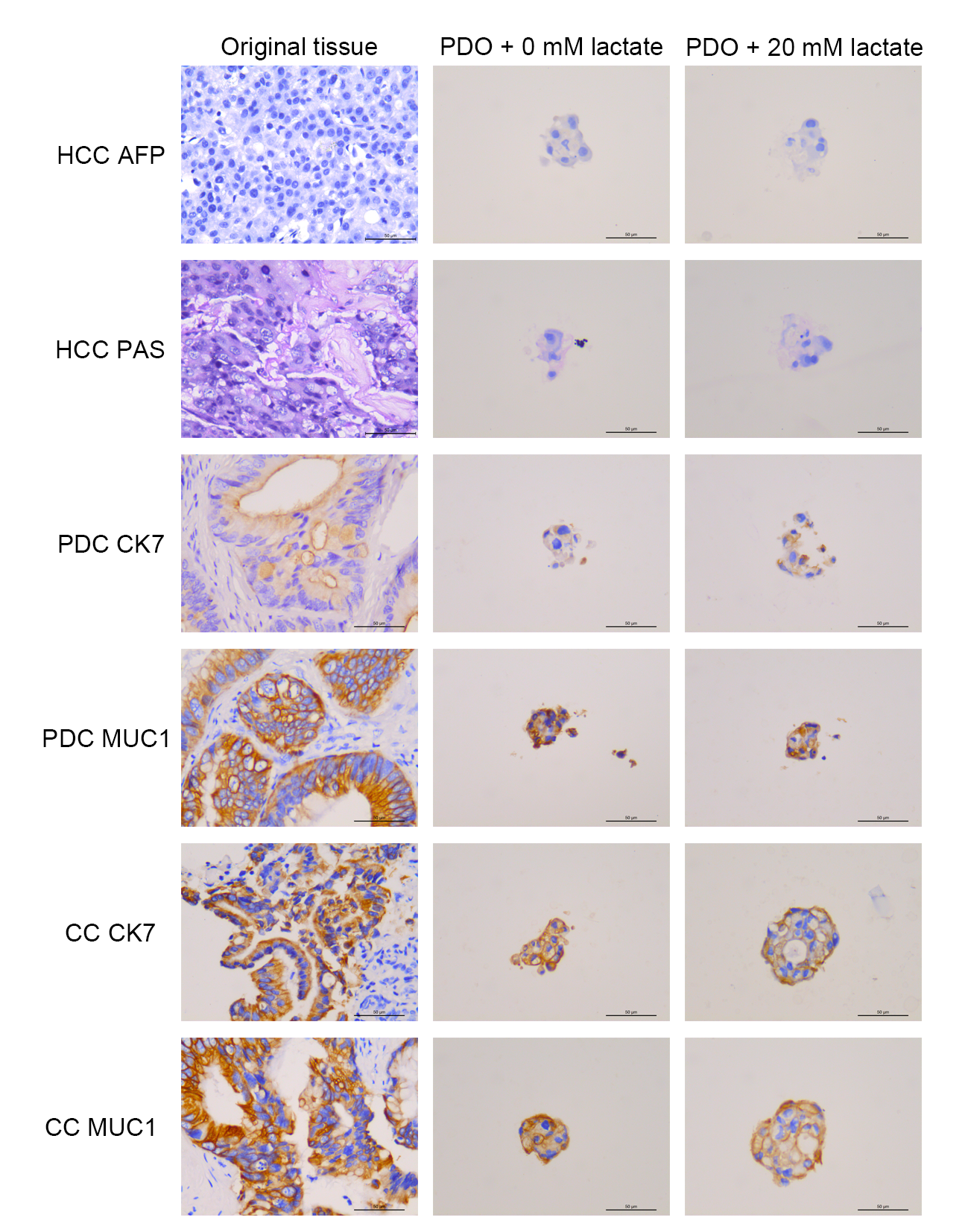

Supplement: Supplementary file 5 — Figure S4 Immunohistochemical staining and PAS staining of parent cancer tissues and cancer PDOs with or without lactate supplementation. [file 41420_2022_1014_MOESM5_ESM.tif]

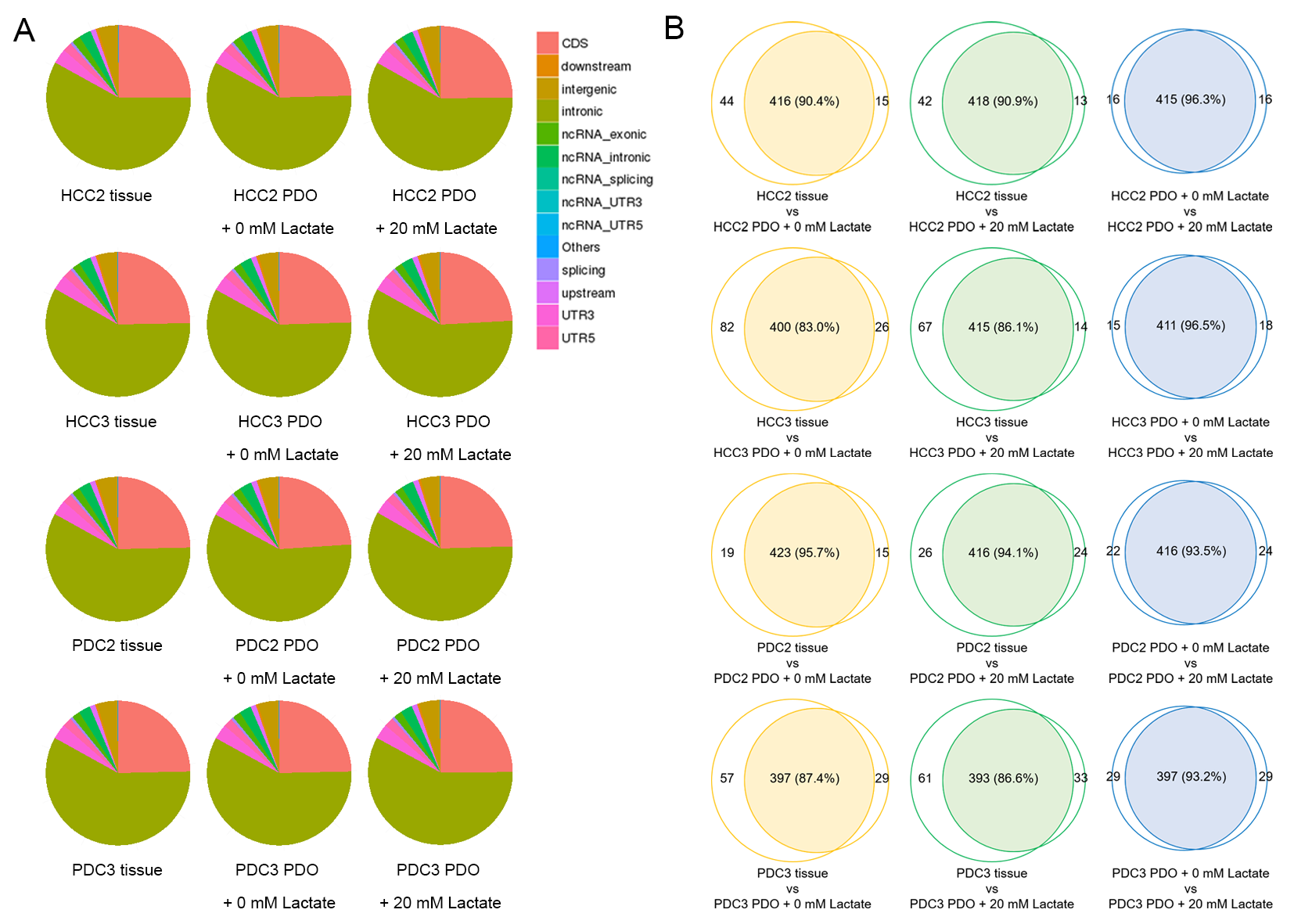

Supplement: Supplementary file 6 — Figure S5 Lactate retained the genetic profiles of PDOs from hepatopancreatobiliary cancers. [file 41420_2022_1014_MOESM6_ESM.tif]

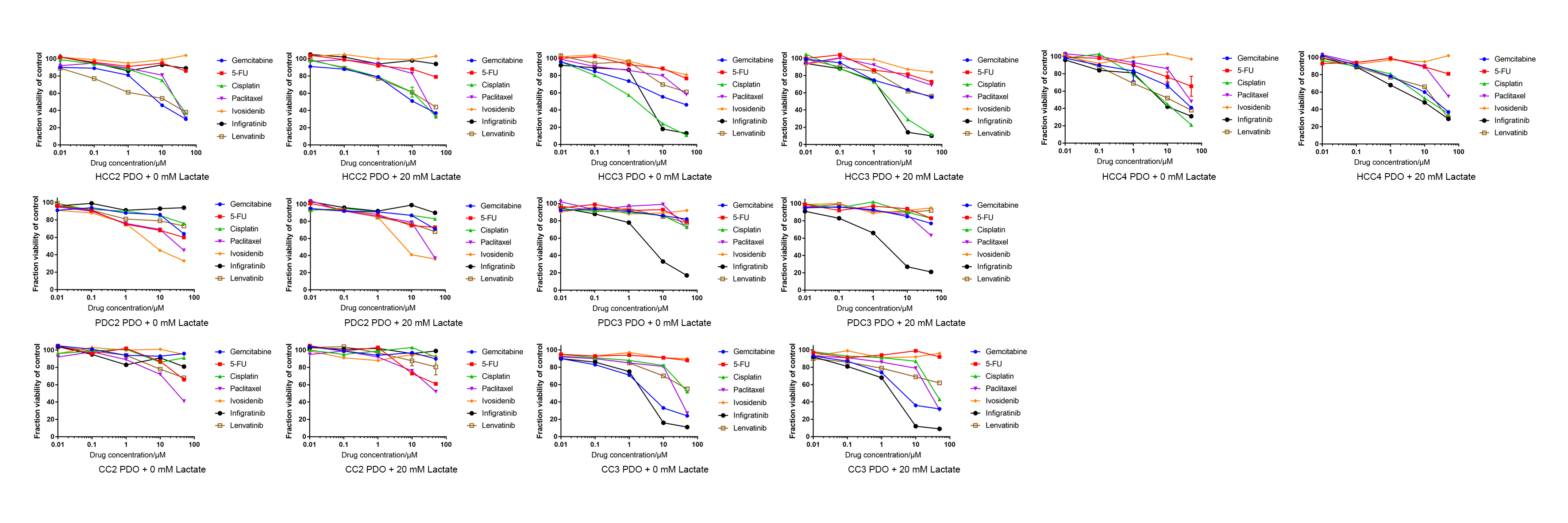

Supplement: Supplementary file 7 — Figure S6 Dose-response curves of HCC2, HCC3, HCC4, PDC2, PDC3, CC2 and CC3 PDOs without or with 20 mM lactate treatment to 7 drugs [file 41420_2022_1014_MOESM7_ESM.tif]

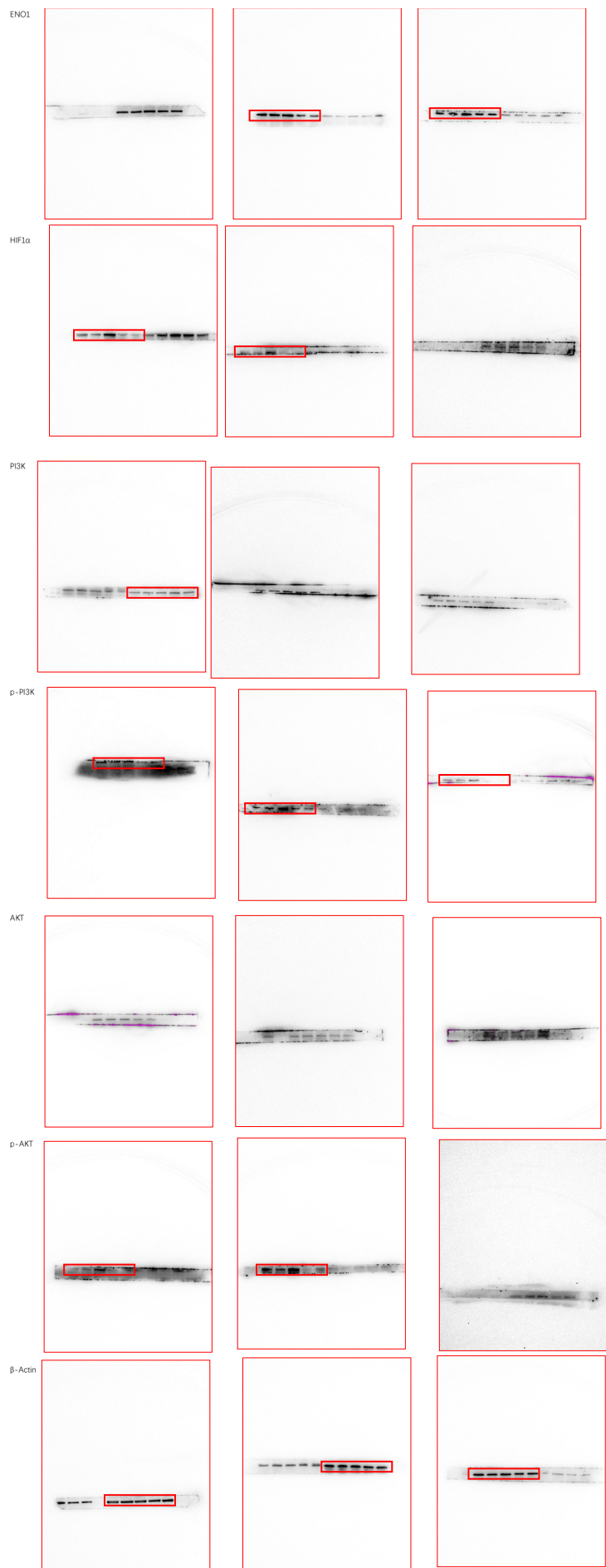

HIF1 $\alpha$

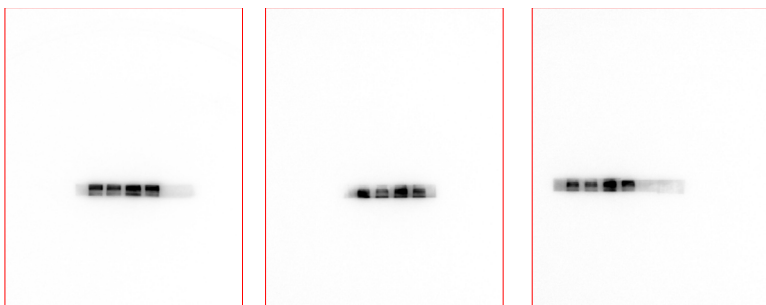

PI3K

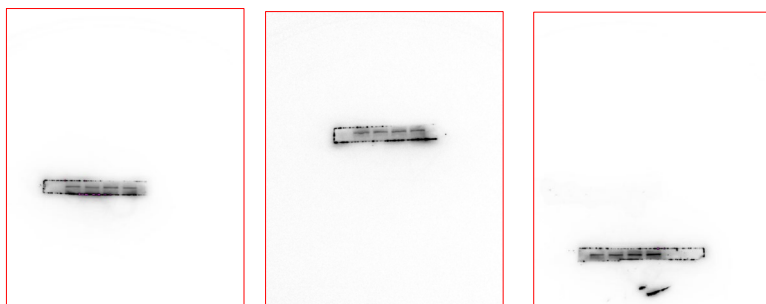

p-PI3K

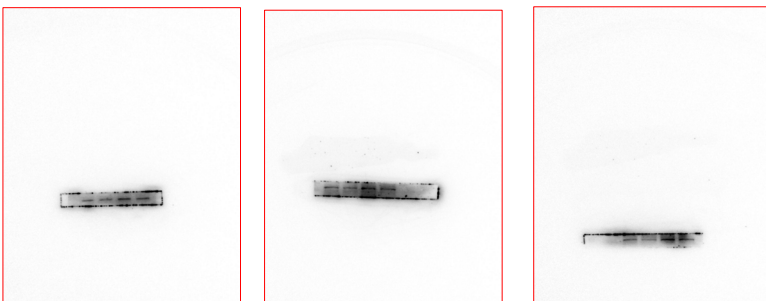

AKT

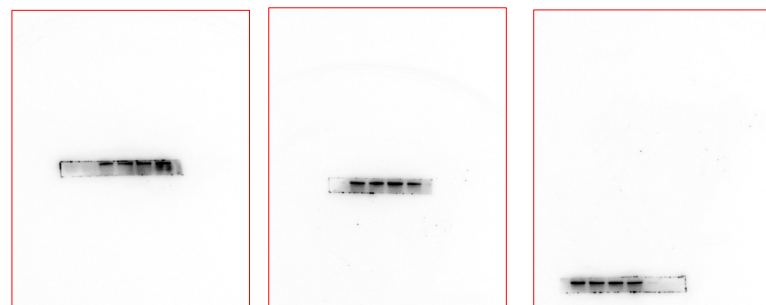

p-AKT

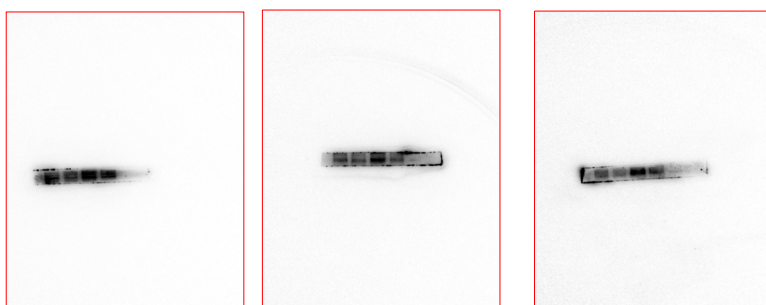

$\beta$ -Actin

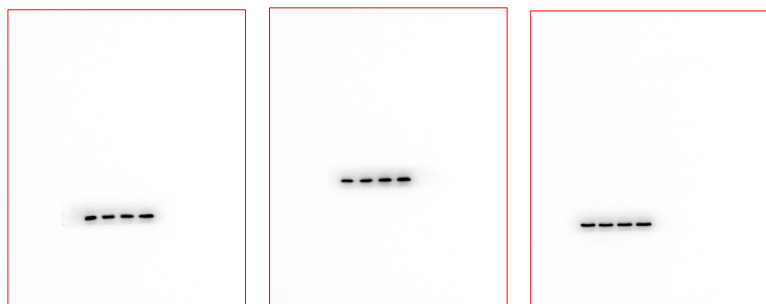

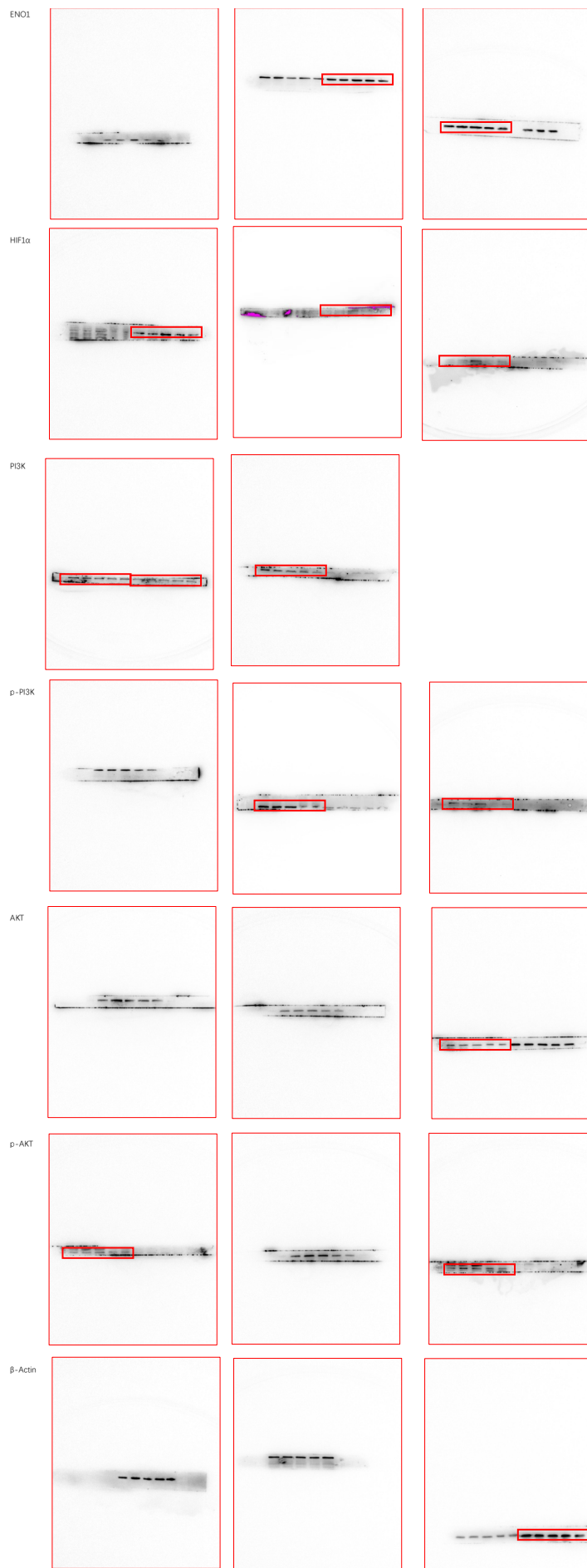

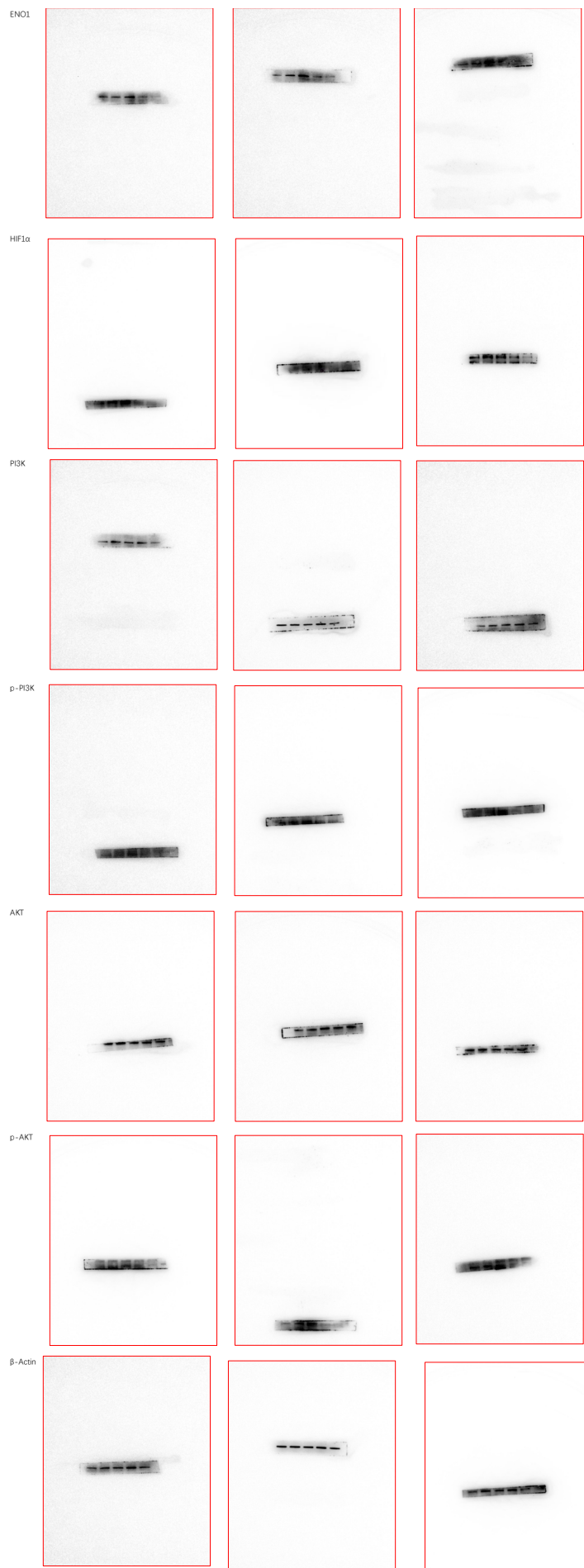

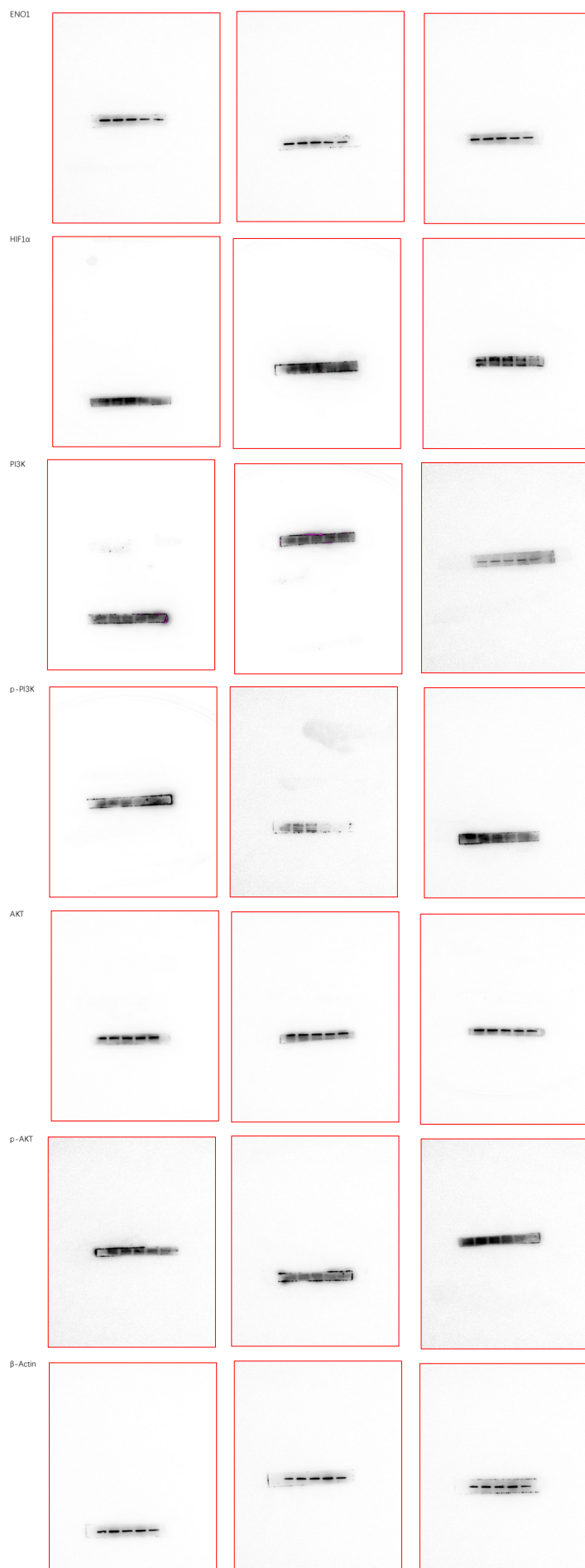

Supplement: Supplementary file 8 — Original western blot [file 41420_2022_1014_MOESM8_ESM.pdf]
